# Evaluation of cycle threshold values at deisolation

**DOI:** 10.1017/ice.2021.132

**Published:** 2021-04-06

**Authors:** Clayton T. Mowrer, Hannah Creager, Kelly Cawcutt, Justin Birge, Elizabeth Lyden, Trevor C. Van Schooneveld, Mark E. Rupp, Angela Hewlett

**Affiliations:** 1 Division of Infectious Diseases, University of Nebraska Medical Center, Omaha, Nebraska; 2 Department of Pathology and Microbiology, University of Nebraska Medical Center, Omaha, Nebraska; 3 Department of Internal Medicine, University of Nebraska Medical Center, Omaha, Nebraska; 4 College of Public Health, University of Nebraska Medical Center, Omaha, Nebraska

## Abstract

The decision to discontinue isolation in hospitalized patients with persistently positive severe acute respiratory coronavirus virus 2 (SARS-CoV-2) molecular testing is nuanced. Improvement in clinical status should be evaluated with expert consultation when considering whether discontinuation of isolation is appropriate. The cycle threshold value may serve as a useful adjunct to this decision-making process.

The emergence of coronavirus disease 2019 (COVID-19), caused by the novel severe acute respiratory syndrome coronavirus-2 (SARS-CoV-2), has evolved into a global pandemic with an unprecedented impact. Due to prolonged shedding of the virus or positivity of nucleic acid amplification tests, particularly in immunocompromised patients or those with severe disease, discontinuation of isolation induces concern for transmission within the healthcare facility, making these decisions nuanced and difficult.^1,2^

Studies evaluating the transmission risk throughout the duration of illness have assessed the growth of SARS-CoV-2 on culture compared with the cycle threshold (Ct) values on RT-PCR. Notably, viable virus is rarely cultured at Ct values >30 on or after 14 days of illness, suggesting that the probability of infectivity decreases with increasing Ct values and is primarily seen in the first 2 weeks after symptom onset.^3,4^ Thus, the Centers for Disease Control and Prevention (CDC) created recommendations for removal from isolation; however, their recommendations regarding immunocompromised persons and those with severe disease were ambiguous.

We implemented a policy in June 2020 allowing discontinuation of isolation in hospitalized patients with COVID-19 21 days after the first positive nucleic acid amplification test (NAAT), stipulating that the COVID-19 infectious diseases team should review each patient prior to discontinuation of isolation (Fig. 1). As a quality improvement initiative, we sought (1) to evaluate how our process for determining isolation discontinuation performed and to (2) describe the clinical characteristics and Ct values of patients approaching 21 days since the first positive test who exhibited persistent viral shedding.

**Fig. 1.**
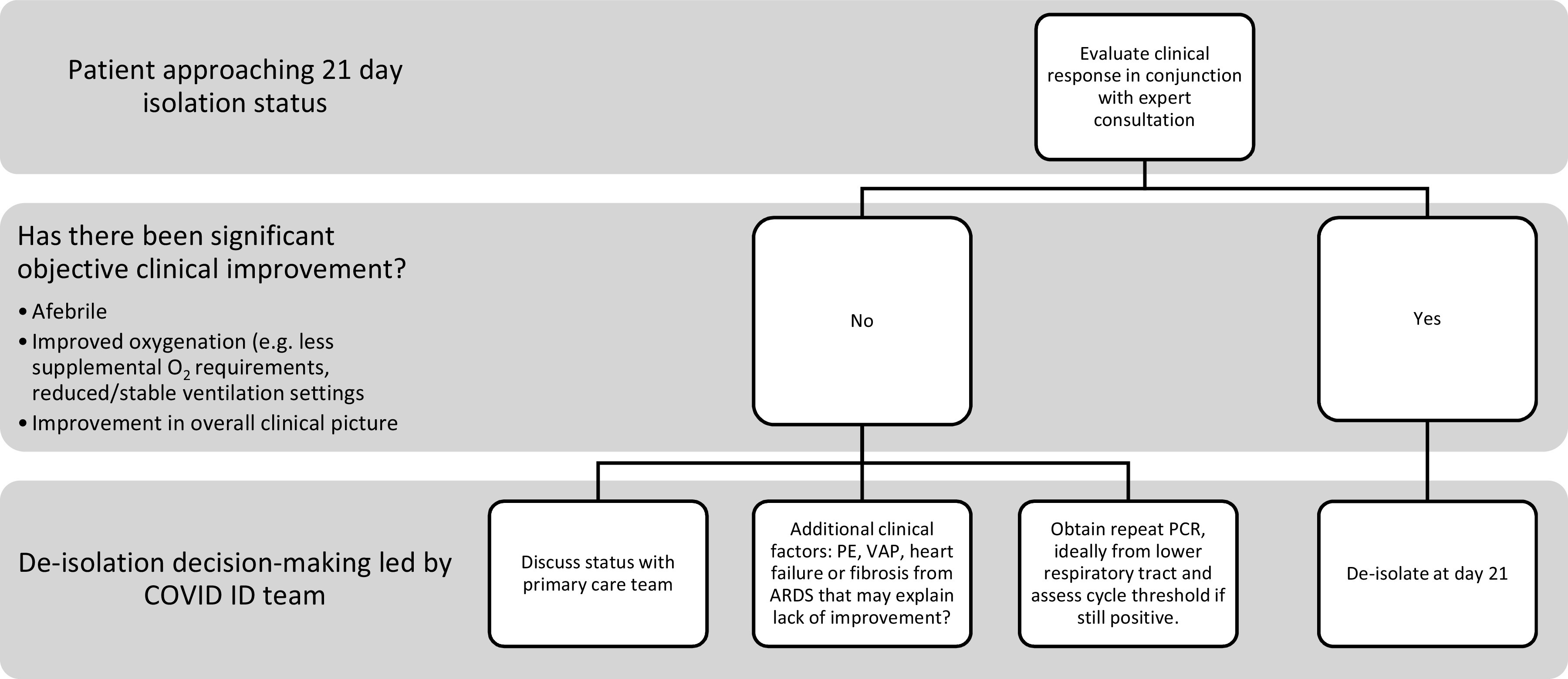
Decision tree for determine if a hospitalized patient is appropriate to remove from isolation. Note. PE, pulmonary embolism; VAP, ventilator-associated pneumonia; ARDS, acute respiratory distress syndrome.

## Methods

We performed a retrospective chart review from June 16, 2020, through September 8, 2020, of adult hospitalized patients with COVID-19 who exhibited persistently positive SARS-CoV-2 NAAT and who were removed from isolation 21 days after their first positive test. Demographics and clinical characteristics were obtained. Immunocompromise was defined as the presence of immunosuppressive medications, advanced HIV, solid-organ or bone-marrow transplantation, or primary immunodeficiency. Severe illness was defined according to NIH criteria.^5^ Only patients who had repeat testing within 21 days were included in the analysis.

COVID testing was performed on multiple assays: the Roche cobas SARS-CoV-2 test, the Cepheid Xpert Xpress SARS-CoV-2, the Hologic Aptima SARS-CoV-2 assay, and the NEcov19 (laboratory-developed with emergency use authorization from the US Food and Drug Administration). Ct values for the E gene, which is targeted by the 3 PCR assays, were obtained and pooled. The Aptima assay utilized transcription-mediated amplification rather than PCR and does not give Ct values.

A linear mixed model that allowed for the correlation of Ct values obtained from the same patient was used to compare the mean Ct values at <10 days, 10–21 days, and ≥21 days from the first positive test. Pairwise comparisons between these periods were adjusted using Tukey’s test. Results were summarized with model adjusted means and standard errors (SEs). Analyses were conducted using SAS version 9.4 software (SAS Institute, Cary, NC), and a *P* value <.05 was considered statistically significant.

## Results

In total, 23 patients (61% male) with persistently positive NAAT testing were removed from isolation after 21 days. Among them, 19 patients had severe disease (83%), 4 (17%) were immunocompromised, and 7 (30%) remained on mechanical ventilation.

**Fig. 2.**
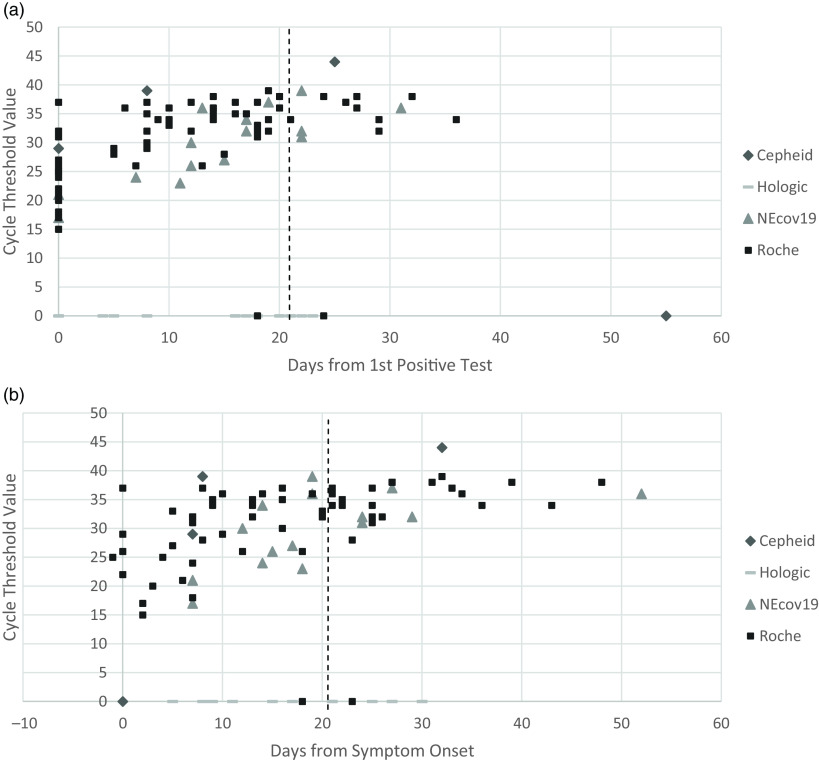
Panel A demonstrates cycle threshold (Ct) values for the SARS-CoV-2 E gene versus days from the first positive test. The vertical line represents day 21, when patients were removed from isolation. Panel B demonstrates Ct values versus days from symptom onset. The vertical line indicates day 21 from symptom onset. *Points on the “0” line represent tests run on Hologic Aptima SARS-CoV-2 assay, which uses transcription-mediated amplification rather than PCR and therefore does not have Ct values, as well as 3 tests in which the N2 target was detected but the E gene target was not.

There were 94 total positive tests among 23 patients: 86 (91.5%) were from nasopharyngeal (NP) samples, 7 (7.4%) were from tracheal aspirates, and 1 (1.1%) was from a bronchoalveolar lavage. The E gene Ct values for each sample are illustrated in Figure 2. Ct values were ≥30 in 39% of tests from days 0–10, in 84% from days 11–21, and in 100% after day 21. Statistically significant differences in the mean Ct value were determined between all 3 periods: <10 days: 26.1 ± 1.1; 10–21 days: 33.1 ± 1.1; >21 days: 38.7 ± 1.5 (*P* < .001).

## Discussion

Patients with COVID-19 may demonstrate prolonged detection of viral RNA for weeks to months after onset of illness, particularly in severe disease.^1^ Studies show that the probability of isolating replication-competent virus in patients is highest at the onset of symptoms and decreases thereafter, with the probability of detection of ˜0.03% by 14 days from symptom onset.^4,6,8^ A correlation between the Ct value and the likelihood of infectivity has been demonstrated.^4,9^ In general, replication-competent virus has not been detected in specimens with Ct values >30, though exceptions have been noted in immunocompromised persons.^3,4,6,10^ However, the comparability of Ct values between different assays and institutions remains unclear.

Removal from isolation at our institution was initially test based: with defervescence and significant clinical improvement, patients could exit isolation with 2 sequential negative molecular tests ∼24 hours apart. This procedure resulted in prolonged hospitalizations for patients who continued to test positive. In June 2020, we implemented a time-based isolation policy allowing the discontinuation of isolation in hospitalized patients 21 days from their first positive test, with the caveat that patients who remained critically ill, lacked clinical improvement, or were severely immunocompromised should be discussed with the COVID ID physician (Fig. 1). Data for patients with severe disease or immunosuppression are limited; thus, decisions to remove these patients from isolation while molecular assays continue to yield positive results are more nuanced. In July 2020, the CDC updated their guidance and recommended a similar strategy based on time from symptom onset. However, we decided to continue our protocol of utilizing the initial positive test as ‘day 1’ given that day of symptom onset is subjective in nature.

Our findings indicate that Ct values were lowest around the time of symptom onset and increased over time thereafter. After day 21 from test positivity, all 14 tests (from 7 patients) showed a Ct value >30 (mean, 31.6), including those from patients with immunosuppression. Additionally, to date, no cases of transmission within our healthcare system have been linked to patients removed from isolation at day 21.

These findings suggest that, when a process is utilized in which clinical criteria are evaluated in conjunction with an infectious disease expert, discontinuation of isolation on day 21 is reasonable and safe. This conclusion is further demonstrated by the higher Ct values and lack of any transmission events following deisolation.

This study has several limitations. Because of the limited study size and because Ct values were obtained from testing performed as part of patient care, data from multiple specimen types and assays were analyzed together. The precision of Ct values obtained from qualitative assays is unknown, and the quality of specimen collection likely provides an additional source of variability. Furthermore, not all patients received testing on or after day 21. Finally, we utilized 21 days from the first positive test rather than time from symptom onset based on our hospital protocol, making this a more conservative discontinuation of isolation strategy. However, only 1 patient had a Ct of <30 when symptom onset was used as day 1, so using symptom onset, although less conservative and more subjective, may also be an adequate strategy.

The decision to discontinue isolation in hospitalized patients with COVID-19 is nuanced, with multiple contributing factors, and it becomes more challenging in patients with severe disease or who are immunocompromised. These decisions should be made with utmost care given concern for transmission within the healthcare facility. Our multistep process, which includes consultation from an expert in infectious diseases and infection control, alongside evaluation of the patient’s medical history, clinical course, and Ct value, is useful when considering whether discontinuation of isolation is appropriate.
